# Stabilisation of the Fatty Acid Decarboxylase from *Chlorella variabilis* by Caprylic Acid

**DOI:** 10.1002/cbic.202100182

**Published:** 2021-06-01

**Authors:** Yinqi Wu, Caroline E. Paul, Frank Hollmann

**Affiliations:** ^1^ Department of Biotechnology Delft University of Technology Van der Maasweg 9 2629HZ Delft The Netherlands

**Keywords:** caprylic acid, fatty acids, fatty acid photodecarboxylase, photochemical inactivation, photostability

## Abstract

The fatty acid photodecarboxylase from *Chlorella variabilis* NC64 A (*Cv*FAP) catalyses the light‐dependent decarboxylation of fatty acids. Photoinactivation of *Cv*FAP still represents one of the major limitations of this interesting enzyme *en route* to practical application. In this study we demonstrate that the photostability of *Cv*FAP can easily be improved by the administration of medium‐chain length carboxylic acids such as caprylic acid indicating that the best way of maintaining *Cv*FAP stability is ‘to keep the enzyme busy’.

## Graphical abstract

**Keep me busy**: The photoactivated fatty acid decarboxylase from *Chlorella variabilis* NC64 A (*Cv*FAP) is a promising catalyst to generate next generation biodiesel from (waste) fatty acids. CvFAP, however is also plagued by pronounced photoinactivation, which can be alleviated by keeping it busy, i. e. converting carboxylic acids such as caprylic acid.

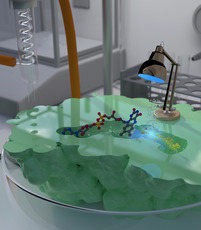

The recently discovered fatty acid photodecarboxylase from *Chlorella variabilis* NC64 A (*Cv*FAP) catalyses the light‐driven decarboxylation of fatty acids into their corresponding (C1‐shortened) alkanes.^[1]^ *Cv*FAP‐catalysed transformations may play a role in the synthesis of fuel alkanes^[2]^ or value‐added fine chemicals.^[3]^ Next to DNA photolyase^[4]^ and protochlorophyllide oxidoreductase,^[5]^ the flavin adenine dinucleotide (FAD)‐containing *Cv*FAP so far represents the only known example of a photoenzyme.

Upon illumination, the photoexcited FAD cofactor (^1^FAD*) inside the enzyme abstracts a single electron from the active site‐bound carboxylate substrate resulting in the FAD semiquinone radical (FAD^•−^) and carboxyl radical. The latter rapidly decarboxylates yielding a short‐lived alkyl radical (R^•^) which abstracts a H‐atom from conserved cysteine or asparagine active site residues. The catalytic cycle is closed by an electron transfer from FAD^•−^ to the amino acid radical (Scheme 1).[^1b^, ^6^]

**Scheme 1:**
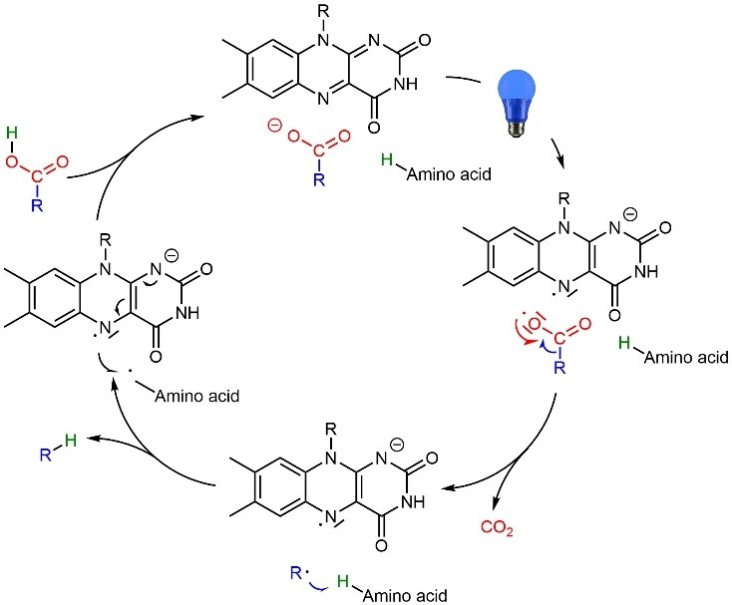
Catalytic cycle of the photoenzymatic decarboxylation of carboxylic acids catalysed by the photodecarboxylase from *Chlorella variabilis* NC64A (*Cv*FAP).

Similar to other flavin‐containing enzymes,^[7]^ *Cv*FAP is prone to photochemical inactivation.^[8]^ Scrutton and co‐workers therefore suggested keeping *Cv*FAP as much as possible under dark (or red light) conditions to minimise its inactivation.^[8]^ The same authors also reasoned that the photoinactivation may originate from a *Cv*FAP malfunction upon photoactivation in the absence of a carboxylate substrate. In this situation, the photoexcited, high redox‐potential flavin is assumed to oxidise nearby active site amino acid residues leading to irreversible inactivation of the enzyme.

We therefore set out to investigate whether the light‐dependent inactivation of *Cv*FAP may simply be alleviated by incubating the enzyme with carboxylic acids.

Prior to investigating the effect of irradiation on the stability of *Cv*FAP, we first tested the thermal stability of the enzyme to rule out possible effect of thermal inactivation on the photoinactivation experiments. *Cv*FAP rapidly lost its catalytic activity upon incubation at temperatures higher than 30 °C (t_1/2_(40 °C)∼2.7 h; t_1/2_(50 °C)<1 h; after 24 h incubation at the temperatures, the catalytic activity was completely lost), whereas at 30 °C, more than 90 % of its initial activity was retained for at least 22 h (Figure 1 and Figure S2). We therefore continued our investigations under conditions at 30 °C.

**Figure 1:**
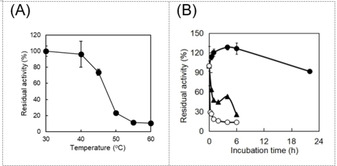
Thermal stability of *Cv*FAP under dark conditions. (A) Residual activity of *Cv*FAP after 15 min incubation at the temperature indicated, (B) Time course of *Cv*FAP activity upon incubation at 30 °C (•), 40 °C (▴), 50 °C (○). Incubation conditions: [*Cv*FAP]=18 μM, buffer: 100 mM Tris‐HCl (pH 8.5), protected from light. Activity assay conditions: [palmitic acid]_0_=13 mM, [DMSO]=30 vol %, buffer: 100 mM Tris‐HCl (pH 8.5), [*Cv*FAP]=3–6 μM, light intensity of blue light=14.5 μE L^−1^ s^−1^, T=37 °C, reaction time=30 min. Data represent the mean±SD of two independent experiments.

Next, we compared the photochemical inactivation of *Cv*FAP as purified enzyme and as crude cell extract preparation (Figure 2). In previous studies we had already observed a significant difference in *Cv*FAP performance in purified form and as crude cell extract.^[2c]^ Under blue light illumination, the crude cell extract preparation of *Cv*FAP was significantly more stable exhibiting a half‐life time of approx. 19 h. Under the same conditions, the purified enzyme was almost completely inactivated within 2 h (t_1/2_∼1 h). Under dark conditions, both enzyme preparations exhibited comparable robustness.

**Figure 2:**
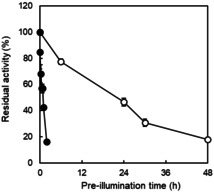
Comparison of photochemical inactivation of *Cv*FAP as purified enzyme (•) and crude cell extract preparation (○). Incubation conditions: [*Cv*FAP]=18 μM, buffer: 100 mM Tris‐HCl (pH 8.5), light intensity of blue light=14.5 μE L^−1^ s^−1^, T=30 °C. Activity assay conditions: [palmitic acid]_0_=13 mM, [DMSO]=30 vol %, buffer: 100 mM Tris‐HCl (pH 8.5), [*Cv*FAP]=3–6 μM, light intensity of blue light=14.5 μE L^−1^ s^−1^, T=37 °C, reaction time=30 min. Data represent the mean±SD of two independent experiments.

It is worth mentioning here that the inactivation of the purified enzyme also depended on the wavelength of the light applied during incubation. Blue light (λ_max_=450 nm) had the most pronounced inactivating effect, followed by green light (λ_max_=550 nm) whereas red light (λ_max_=650 nm) slightly influenced the stability of purified *Cv*FAP (Figure 3). This corresponds well with the UV/Vis spectrum of *Cv*FAP‐bound FAD and supports the assumption of photoexcited FAD being the main cause of photoinactivation.

**Figure 3:**
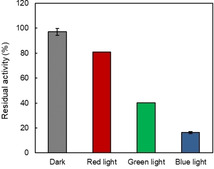
Residual activity of purified *Cv*FAP illuminated under different wavelength of LEDs. Incubation conditions: [*Cv*FAP]=18 μM, buffer: 100 mM Tris‐HCl (pH 8.5), protected from light or illuminated by different colour of light, T=30 °C, pre‐illumination time=2 h. Activity assay conditions: [palmitic acid]_0_=13 mM, [DMSO]=30 vol %, buffer: 100 mM Tris‐HCl (pH 8.5), [*Cv*FAP]=3–6 μM, light intensity of blue light=14.5 μE L^−1^ s^−1^, T=37 °C, reaction time=30 min. Data represent the mean±SD of two independent experiments.

The strikingly higher photostability of *Cv*FAP in crude cell extracts (Figure 2) may be attributed to the presence of *E. coli*‐borne carboxylic acids in these preparations. We therefore examined the influence of various carboxylic acids on the photostability of purified *Cv*FAP (Figure 4).

**Figure 4:**
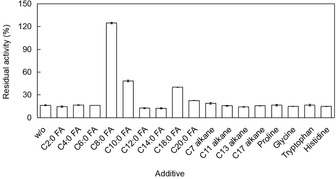
Influence of additive on the photostability of purified *Cv*FAP. Incubation condition: [*Cv*FAP]=18 μM, buffer: 100 mM Tris‐HCl (pH 8.5), [DMSO]=5 vol %, light intensity of blue light=14.5 μE L^−1^ s^−1^, T=30 °C, pre‐illumination time=2 h, [additive]=10 mM. Activity assay conditions: [palmitic acid]_0_=13 mM, [DMSO]=30 vol %, buffer: 100 mM Tris‐HCl (pH 8.5), [*Cv*FAP]=3–6 μM, light intensity of blue light=14.5 μE L^−1^ s^−1^, T=37 °C, reaction time=30 min. Data represent the mean±SD of two independent experiments.

Alkanes (as reaction products) did not exceed a significant stabilising effect on illuminated *Cv*FAP, whereas several fatty acids stabilised the illuminated enzyme. This indicates that the photostability of *Cv*FAP is linked to its decarboxylation reaction. Interestingly, caprylic acid had the most pronounced stabilising effect. This was somewhat unexpected as according to the previously determined substrate scope of *Cv*FAP,^[1a]^ caprylic acid should be a much poorer substrate compared to C_16_−C_18_ carboxylic acids. Currently, we are lacking a plausible explanation for why octadecanoic/eicosanoic acid were less efficient than caprylic acid, possibly the concentration of free octadecanoic/eicosanoic acid (more hydrophobic and hence exhibiting a lower critical micelle concentration) was lower than the one of caprylic acid. Possibly, the stabilising effect of caprylic acid may also be associated to the ‘decoy effect’ of the C_7_ alkane product.^[2b]^

We further investigated the concentration‐dependency of the stabilising effect of caprylic acid (Figure 5). Increasing the concentration of caprylic acid up to 10 mM steadily increased the stabilising effect, which may well be explained with increasing saturation of the active site of *Cv*FAP. Further increase of the caprylic acid concentration apparently gradually decreased the stabilising effect. Most likely, this, however, is an artefact from the activity assay based on the accumulation of pentadecane. At high caprylic acid concentrations, its decarboxylation competes with the decarboxylation of palmitic acid and thereby reduce the pentadecane formation rate. In fact, caprylic acid could be converted during the pre‐illumination process catalysed by *Cv*FAP. After 24 h pre‐illumination, little caprylic acid was detected and *Cv*FAP exhibited little residual activity, compared with 46 % residual activity of that incubated in dark for 24 h (Figure 2 and Figure S3).

**Figure 5:**
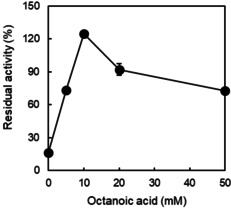
Influence of additive on the photostability of purified *Cv*FAP. Incubation condition: [*Cv*FAP]=18 μM, buffer: 100 mM Tris‐HCl (pH 8.5), [DMSO]=5 vol %, light intensity of blue light=14.5 μE L^−1^ s^−1^, T=30 °C, incubation time=2 h, [caprylic acid]=0‐50 mM. Activity assay conditions: [palmitic acid]_0_=13 mM, [DMSO]=30 vol %, buffer: 100 mM Tris‐HCl (pH 8.5), [*Cv*FAP]=3 μM, light intensity of blue light=14.5 μE L^−1^ s^−1^, T=37 °C, reaction time=30 min. Data represent the mean±SD of two independent experiments.

Finally, we confirmed the stabilising effect of caprylic acid on purified, pre‐illuminated *Cv*FAP used in semi‐preparative transformation of palmitic acid. Figure 6 impressively demonstrates the positive effect of simple caprylic acid in the pre‐illumination reaction. While in the absence of caprylic acid, very low concentration of pentadecane was detected, the product accumulation rate of the caprylic acid protected *Cv*FAP was comparable to activities previously observed (without pre‐illumination).

**Figure 6:**
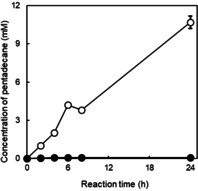
Photoenzymatic conversion of palmitic acid to pentadecane catalysed by pre‐illuminated purified *Cv*FAP. Reaction condition: [*Cv*FAP]=3 μM, pre‐illumination time=2 h, [palmitic acid]_0_=13 mM, [DMSO]=30 vol %, buffer: 100 mM Tris‐HCl (pH 8.5), light intensity of blue light=14.5 μE L^−1^ s^−1^, T=30 °C, [caprylic acid]=10 mM (○) or 0 mM (•). Data represent the mean±SD of two independent experiments.

Photoinactivation of *Cv*FAP remains a major limitation to preparative application of this promising catalyst. In accordance with the *Cv*FAP inactivation mechanism proposed by Scrutton and co‐workers,^[8]^ our results indicate that photoexcitation of *Cv*FAP in the absence of a convertible substrate (carboxylic acid) represents the main cause for *Cv*FAP photoinactivation. In this situation, the photoexcited flavin oxidises active‐site borne amino acids, which in light of the recent mechanistic studies by Beisson and co‐workers,^[1b]^ may be the catalytically active cysteine or arginine (Scheme 2).

**Scheme 2:**
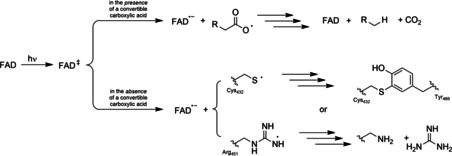
Possible mechanisms of *Cv*FAP inactivation. In the presence of a carboxylic acid substrate the photoexcited flavin prosthetic group catalysed the radical decarboxylation reaction (see also Scheme 1). In the absence of a carboxylic acid, the photoexcited flavin may interact with amino acid residues such as Cys432 or Arg451 (and/or others) to resulting in chemically modified active site amino acid residues.

In the case of cysteine, it may be argued that the thiyl radical (lacking suitable reaction partners) may react with further amino acids such as tyrosine 466. It is also conceivable that arginine 451 undergoes deguanidination. These inactivation reactions may be expected to be dependent on the protonation stage of the amino acids and that pH‐dependent inactivation experiments may shed a light on this. Moreover, digestion of the photoinactivated enzyme and mass‐spectroscopic analysis of the fragments will shed a light on the inactivation mechanism and possibly serve as guiding principle for future *Cv*FAP engineering to increase its photorobustness. Evidently, the generally observed ‘simple’ photobleaching of the flavin prosthetic group may also considerably contribute to the photoinactivation of *Cv*FAP.

In the present study we have shown that the photostability issue of *Cv*FAP, at least under illumination conditions, can significantly be alleviated by the addition of caprylic acid (and possibly some other carboxylic acids not tested yet). In line with previous suggestions by Scrutton and co‐workers, these results point towards ‘keeping *Cv*FAP catalytically busy’ as the most promising strategy to minimise photoinactivation of the enzyme.

## Conflict of interest

The authors declare no conflict of interest.

## Supporting information

As a service to our authors and readers, this journal provides supporting information supplied by the authors. Such materials are peer reviewed and may be re‐organized for online delivery, but are not copy‐edited or typeset. Technical support issues arising from supporting information (other than missing files) should be addressed to the authors.

Supplementary
