# Supplementary material for: Stabilisation of the Fatty Acid Decarboxylase from Chlorella variabilis by Caprylic Acid
Source: Chembiochem. 2021 Jun 1;22(14):2420–3. doi: 10.1002/cbic.202100182 (PMC8362199; doi:10.1002/cbic.202100182)
Supplement: Supplementary file 1 — Supplementary [file CBIC-22-2420-s001.pdf]

ChemBioChem

Supporting Information

Stabilisation of the Fatty Acid Decarboxylase from *Chlorella variabilis* by Caprylic Acid

Yinqi Wu, Caroline E. Paul, and Frank Hollmann*

Contents

1. General Remarks 2

1.1 Materials 2

1.2 Protein expression and purification 2

1.3 Activity assay 2

1.4 Thermal stability assay 3

1.5 Photostability assay 3

1.6 Photoenzymatic decarboxylation of palmitic acid to pentadecane 3

1.7 Light intensity measurement 3

2. GC analyses 4

Table S1. GC column oven programme and retention times of compounds. 4

3. Supporting figures 5

3.1 Production and purification of *Cv*FAP 5

Figure S1. SDS-PAGE analysis of the cell free extract and purified samples of *Cv*FAP during the protein purification process. 5

3.2 Influence of temperature on purified *Cv*FAP-catalysed decarboxylation reaction of palmitic acid to pentadecane 5

Figure S2. Decarboxylation of palmitic acid to pentadecane catalyzed by purified *Cv*FAP at 30 °C () or 37 °C () under blue light illumination. 5

3.3 Stability of purified *Cv*FAP under different conditions .... **Error! Bookmark not defined.**

Figure S3. Time course of stability (expressed as % residual activity) of purified *Cv*FAP illuminated under blue light () or protected from light source (). .... **Error! Bookmark not defined.**

Figure S4. GC chromatograms of the palmitic acid decarboxylation catalyzed by purified *Cv*FAP for 0.5 h which has been pre-illuminated under blue light with 10 mM caprylic acid for 2 h **(A)** and 24 h **(B)**. 6

3.4 Influence of different wavelength light on photostability of *Cv*FAP crude cell extract preparation.. 6

Figure S5. Photostability 6

3.5 Photoreaction setup. 7

Figure S6. The homemade photoreactor setup employed in this study. 7

1. General Remarks

1.1 Materials

Eicosanoic acid (C20:0 FA), octadecanoic acid (C18:0 FA), hexadecanoic acid (C16:0 FA), myristic acid (C14:0 FA), lauric acid (C12:0 FA), decanoic acid (C10:0 FA), caprylic acid (C8:0 FA), hexanoic acid (C6:0 FA), butyric acid (C4:0 FA), acetic acid (C2:0 FA), proline, glycine, tryptophan, histidine, DMSO, heptane (C7 alkene), undecane (C11 alkene), tridecane (C13 alkene), pentadecane (C15 alkene), heptadecane (C17 alkene), bovine serum albumin (BSA) and other commercial chemicals were purchased from Sigma-Aldrich, Fluka, Acros or Alfa-Aesar, without any further purification. The water used was distilled.

1.2 Protein expression and purification

The *E. coli* BL21(DE3) cells containing the plasmid pET-28a(+) expressing *Cv*FAP were cultivated in terrific broth (TB) medium containing 50 μg mL^{-1} kanamycin at 37 °C and 180 rpm. When the optical density at 600 nm (OD_{600}) reached 0.7-0.8, protein induction was initiated by adding 0.5 mM IPTG and cultivation temperature was decreased to 17 °C. After cultivation for about 20 h, cells were harvested by centrifugation (11000 × *g* at 4 °C for 10 min), washed twice with ice-cooled buffer A (50 mM Tris-HCl, 300 mM NaCl, 10 mM imidazole, 10% glycerol, pH 8.0). The cell pellet was resuspended in the same buffer containing 1 mM PMSF and 1 mM $MgCl_2$ and then cells were lysed by passing them through a Multi Shot Cell Disruption System at 1.5 kbar. The lysates were centrifuged at 38000 × *g* at 4 °C for 1 h and then passed through a filter of 0.45 μm to remove the particulate fraction. The purification was made on a His Trap Ni-NTA FF column (5 mL, GE Healthcare). After loading the lysate, the column was washed by 20 vol % buffer B (50 mM Tris-HCl, 300 mM NaCl, 200 mM imidazole, 10% glycerol, pH 8.0) and protein was then eluted by a step gradient using 40 vol% buffer B. The fractions were determined by SDS-PAGE and concentrated by ultrafiltration (50 kDa filters). The Ni-NTA column purification was performed by NGC system in the 10 °C fridge covered by aluminum foil. The concentrated purified protein was loaded on the desalting column (6 mL, PD10) to remove imidazole. Protein was eluted by buffer (100 mM Tris-HCl, pH 8.5). The yellow fractions were collected and protein concentration used for activity assay corresponded the protein containing FAD. FAD was quantified by measuring absorbance at 450 nm on the protein which was previously heated at 95 °C for 5 min in the addition of 1 w/w % SDS.

1.3 Activity assay

The activity of enzyme *Cv*FAP was assayed at 37 °C by monitoring the increase of pentadecane in a 1-mL reaction by using the gas chromatography (Shimadzu GC-2014) equipped with the column CP Sil 5 CB (50 m × 0.53 mm × 1.0 μm), using flame ionization detection (FID), and N_2 as the carrier gas. The standard assay mixture was composed of 13 mM palmitic acid as substrate, 30 vol % DMSO as cosolvent, buffer (100 mM Tris-HCl, pH 8.5), and *Cv*FAP with an appropriate concentration and was under gentle magnetic stirring at 37 °C under the illumination of blue light (light intensity=14.5 μE L^{-1} s^{-1}) for 30 min.

1.4 Thermal stability assay

The residual activity of CFE *Cv*FAP and purified *Cv*FAP were determined by incubating the enzyme (18 μM) in buffer (100 mM Tris-HCl, pH 8.5) at different temperature protected from light for a proper time and then performed the activity assay. The activity of *Cv*FAP without any incubation before the activity assay was defined as 100% residual activity.

1.5 Photostability assay

The residual activity of CFE *Cv*FAP and purified *Cv*FAP were determined by incubating the enzyme *Cv*FAP (18 μM) in buffer (100 mM Tris-HCl, pH 8.5) at 30 °C under the illumination of LEDs for a proper time and then performed the activity assay. The activity of *Cv*FAP without any incubation or pre-illumination before the activity assay was defined as 100% residual activity. The half-lives ($t_{1/2}$) of enzyme under corresponding conditions were calculated according to the deactivation function: ln(residual activity)=-k_D/t; $t_{1/2}$=$ln2/k_D$. k_D here represents the deactivation rate constant.

1.6 Photoenzymatic decarboxylation of palmitic acid to pentadecane

The 1 mL reaction was composed of 13 mM palmitic acid as substrate, 30 vol % DMSO as cosolvent, 3 μM purified *Cv*FAP, and buffer (100 mM Tris-HCl, pH 8.5) and was under gentle magnetic stirring at 30 °C under the illumination of blue light (light intensity=14.5 μE L^{-1} s^{-1}). Samples were withdrawn and extracted with ethyl acetate (containing 5 mM 1-octanol) for gas chromatography analysis.

1.7 Light intensity measurement

The light intensity was determined by means of ferrioxalate actinometry. 1 mL ferrioxalate solution (37.5 mM in 50 mM H_2SO_4) in a 4-mL transparent glass vial was illuminated under LED light. At defined intervals, 25 µL samples of the illuminated solution were taken and mixed with 175 µL of another solution (7.5 mL 50 mM H_2SO_4, 2 mL 0.1% 1,10-phenantroline, 5 mL 1 M sodium acetate solution and 3 mL H_2O). The absorbance of the mixture was measured under 510 nm at room temperature. $FeSO_4$ was used as Fe(II) for the calibration curve. The light intensity was then calculated based on the Fe(II) generation rate in the irradiated ferrioxalate solution.

2. GC analyses

The enzyme activity and time course of decarboxylation reaction catalyzed by *Cv*FAP were measured by monitoring the production of pentadecane by using the gas chromatography (Shimadzu GC-2014) with FID, equipped with column CP Sil 5 CB (50 m × 0.53 mm × 1.0 μm), 20 mL/min N_2 as the carrier gas. The injection temperature was 340 °C.

Table S1. GC column oven programme and retention times of compounds.

Compound	Retention time (min)	Temperature profile
Palmitic acid (substrate)	10.76	110 °C hold 3 min, 25 °C/min to 190 °C hold 2.1 min, 25 °C/min to 230 °C hold 2.1 min, 30 °C/min to 325 °C hold 1 min
1-Octanol (internal standard)	3.23	
Pentadecane (product)	6.79	
Caprylic acid (additive)	4.26	

3. Supporting figures

3.1 Production and purification of *Cv*FAP

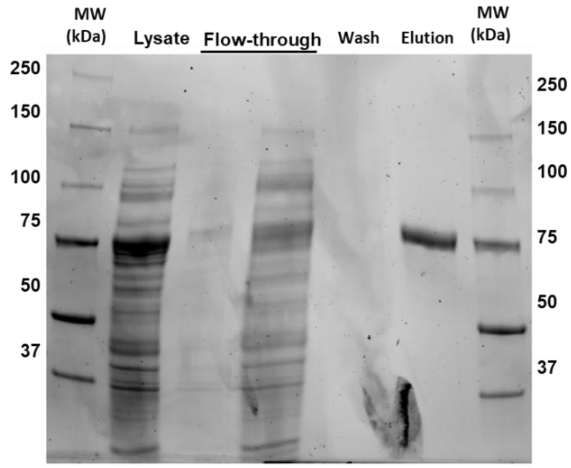

Figure S1. SDS-PAGE analysis of the cell free extract and purified samples of *Cv*FAP during the protein purification process. From left to right: (1) Molecular weight marker; (2) CFE *Cv*FAP; (3) Flow-through from the Ni-column; (4) Wash fractions; (5) Elution fractions; (6) Molecular weight marker. The apparent subunit molecular mass of *Cv*FAP is 77 kDa.

3.2 Influence of temperature on purified *Cv*FAP-catalysed decarboxylation reaction of palmitic acid to pentadecane

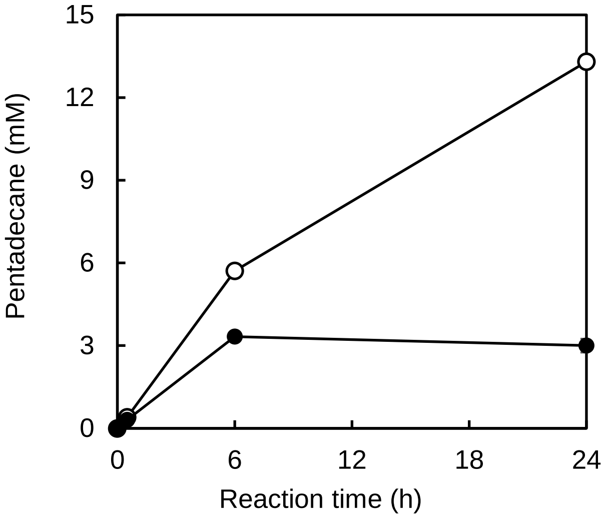

Figure S2. Decarboxylation of palmitic acid to pentadecane catalyzed by purified *Cv*FAP at 30 °C (○) or 37 °C (●) under blue light illumination. Reaction condition: [*Cv*FAP]=3 μM, [palmitic acid]$_0$=13 mM, [DMSO]=30 vol %, buffer: 100 mM Tris-HCl (pH 8.5), light intensity of blue light=14.5 μE L^{-1} s^{-1}, T=30 °C or 37 °C. Data represent the mean ± SD of two independent experiments.

(A)

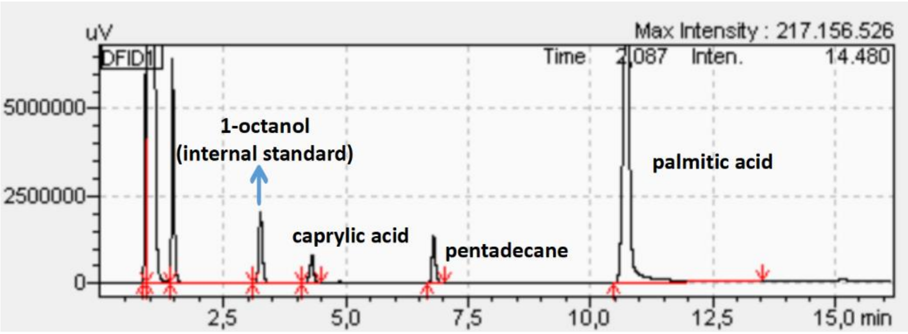

(B)

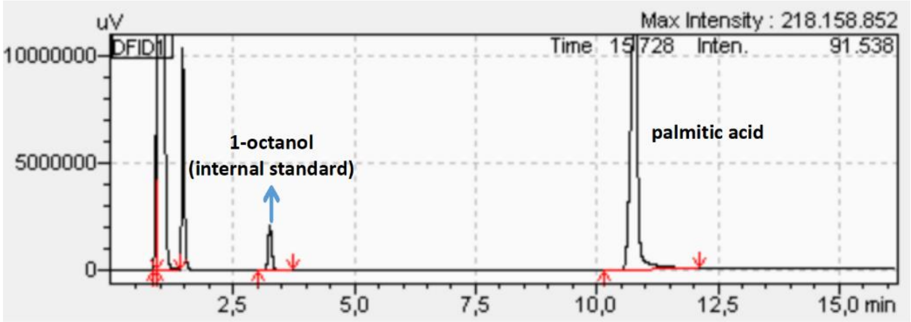

Figure S3. GC chromatograms of the palmitic acid decarboxylation catalysed by purified *Cv*FAP for 0.5 h which has been pre-illuminated under blue light with 10 mM caprylic acid for 2 h **(A)** and 24 h **(B)**.

3.4 Influence of different wavelength light on photostability of *Cv*FAP crude cell extract preparation

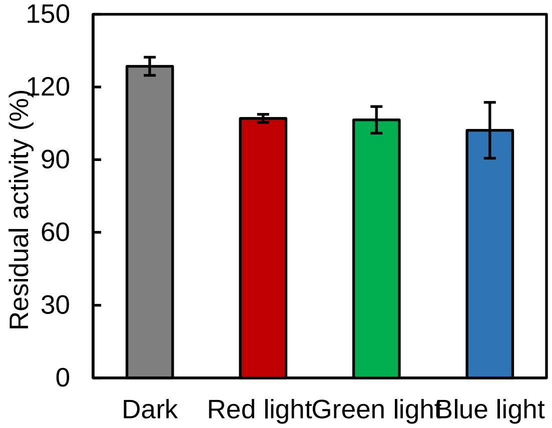

Figure S4. Photostability (expressed as % residual activity) of *Cv*FAP crude cell extract preparation illuminated under different wavelength of LEDs. Incubation condition: [*Cv*FAP]=18 µM, buffer: 100 mM

Tris-HCl (pH 8.5), under illumination of different wavelength of light, T= 30 °C, incubation time=4 h. Data represent the mean ± SD of two independent experiments.

3.5 Photoreaction setup.

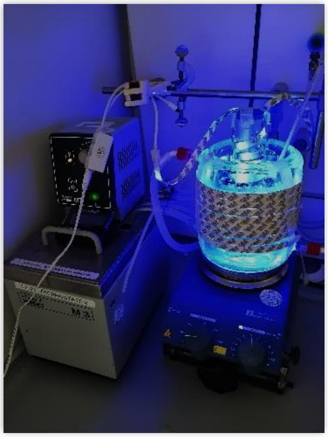

Figure S5. The homemade photoreactor setup employed in this study.
